# Oridonin Confers Protection against Arsenic-Induced Toxicity through Activation of the Nrf2-Mediated Defensive Response

**DOI:** 10.1289/ehp.11464

**Published:** 2008-05-21

**Authors:** Yu Du, Nicole F. Villeneuve, Xiao-Jun Wang, Zheng Sun, Weimin Chen, Jixue Li, Hongxiang Lou, Pak Kin Wong, Donna D. Zhang

**Affiliations:** 1 Department of Pharmacology and Toxicology, College of Pharmacy, University of Arizona, Tucson, Arizona, USA; 2 Department of Natural Products, School of Pharmaceutical Sciences, Shandong University, Shandong, People’s Republic of China; 3 Aerospace and Mechanical Engineering Department, University of Arizona, Tucson, Arizona, USA

**Keywords:** antioxidant responsive element, antitumor, ARE, arsenic, chemoprevention, diterpenoid, Keap1, Nrf2, oridonin, oxidative stress, rubescensin

## Abstract

**Background:**

Groundwater contaminated with arsenic imposes a big challenge to human health worldwide. Using natural compounds to subvert the detrimental effects of arsenic represents an attractive strategy. The transcription factor nuclear factor erythroid 2-related factor 2 (Nrf2) is a critical regulator of the cellular antioxidant response and xenobiotic metabolism. Recently, activation of the Nrf2 signaling pathway has been reported to confer protection against arsenic-induced toxicity in a cell culture model.

**Objectives:**

The goal of the present work was to identify a potent Nrf2 activator from plants as a chemopreventive compound and to demonstrate the efficacy of the compound in battling arsenic-induced toxicity.

**Results:**

Oridonin activated the Nrf2 signaling pathway at a low subtoxic dose and was able to stabilize Nrf2 by blocking Nrf2 ubiquitination and degradation, leading to accumulation of the Nrf2 protein and activation of the Nrf2-dependent cytoprotective response. Pretreatment of UROtsa cells with 1.4 μM oridonin significantly enhanced the cellular redox capacity, reduced formation of reactive oxygen species (ROS), and improved cell survival after arsenic challenge.

**Conclusions:**

We identified oridonin as representing a novel class of Nrf2 activators and illustrated the mechanism by which the Nrf2 pathway is activated. Furthermore, we demonstrated the feasibility of using natural compounds targeting Nrf2 as a therapeutic approach to protect humans from various environmental insults that may occur daily.

Arsenic is a major environmental pollutant that exists in soil and minerals; it readily enters the groundwater system, contaminating drinking water. The concentration of arsenic in ground-water varies significantly in different geographic areas. Arsenic concentrations are highest in East Asia, including Bangladesh; West Bengal, India; and China (Kumagai and Sumi 2007; Smith et al. 2000; Tchounwou et al. 2003). Many efforts have been made to reduce arsenic damage as exemplified by the guideline for arsenic in drinking water set by the World Health Organization (Smith and Smith 2004) and by local governments. Nevertheless, a large number of populations are still at risk of arsenic exposure and are suffering from arsenic-induced adverse effects, such as hypertension, arteriosclerosis, diabetes, hyperkeratosis, neuropathy, and skin, liver, bladder, and lung cancer (Kumagai and Sumi 2007; Smith et al. 2006; Steinmaus et al. 2000; Tseng 2002). Clearly, the best way to protect humans from arsenic-induced damage is to reduce arsenic intake. However, it is not always practical because many people have no choice but to consume drinking water and rice heavily contaminated with arsenic, as these are their only sources of food and water. Therefore, an alternative choice, of equal importance, is to subvert the detrimental effects of arsenic by modulating the body’s defense system.

Nuclear factor erythroid 2-related factor 2 (Nrf2) is a critical transcription factor that regulates a cytoprotective response. Many of its downstream target genes are important in maintaining the cellular antioxidant response and xenobiotic metabolism. For example, γ-glutamylcysteine synthetase (GCS) and the xCT cysteine antiporter are the key enzymes for synthesis of glutathione and maintenance of cellular redox homeostasis (Chan and Kwong 2000; Sasaki et al. 2002; Wild et al. 1999). Conjugating enzymes, such as glutathione *S*-transferases (GSTs) and UDP-glucuronosyl transferase, facilitate the removal of toxic and carcinogenic chemicals by increasing their solubility and excretion (Kobayashi and Yamamoto 2006; Zhang 2006). Many transporters such as multidrug resistance proteins and p-glycoprotein are important in uptake and removal of xenobiotics (Hayashi et al. 2003; Maher et al. 2005; Vernhet et al. 2001; Xu et al. 2005). Activation of the Nrf2 signaling pathway is tightly regulated by Kelch-like ECH-associated protein 1 (Keap1) according to changes in the intracellular redox state when cells are exposed to exogenous stimuli. Under normal conditions, cells maintain low constitutive levels of Nrf2-target genes through constant ubiquitination and degradation of Nrf2, which is accomplished by the Keap1-dependent E3 ubiquitin ligase complex. Upon induction, Nrf2 is stabilized because of impaired Keap1-E3 ubiquitin ligase activity, which results in activation of the Nrf2 signaling pathway (Cullinan et al. 2004; Furukawa and Xiong 2005; Kobayashi et al. 2004; Sun et al. 2007; Zhang et al. 2004b). Chemopreventive compounds are able to activate the Nrf2-dependent adaptive response and thus confer protection against subsequent toxic or carcinogenic damage (Jeong et al. 2006; Yates and Kensler 2007).

In addition to the beneficial antioxidants and many chemopreventive compounds, the Nrf2 signaling pathway can also be induced by many harmful chemicals such as arsenic, hydrogen peroxide, and even anticancer drugs including cisplatin (Aono et al. 2003; He et al. 2006; Massrieh et al. 2006; Pi et al. 2003; Purdom-Dickinson et al. 2007; Wang et al. 2006). This paradox may be explained by the balance between the induction of the Nrf2 defensive response and the toxic outcome elicited by a particular compound. The most attractive chemopreventive compounds are those that potentially induce the Nrf2-dependent defensive response without eliciting toxic effects, that is, those that tip the balance toward the Nrf2-dependent beneficial response. In accordance with this notion, many chemopreventive compounds extracted from dietary sources or plants activate the Nrf2-dependent response at low doses and do not elicit detectable toxic effects. Nrf2 activators identified so far can be classified into categories that include phenolic antioxidants (caffeic acid, epigallocatechin-3-gallate, butylated hydroxyanisole), dithiolethiones (oltipraz, 3H-1,2-dithiole-3-thione), isothiocyanates (sulforaphane), and triterpenoids [1-(2-cyano-3,12-dioxooleane-1,9[11]-dien-28-oyl)imidozole] (Yates and Kensler 2007; Zhang 2006). Up-regulation of the Nrf2-dependent defense response has proved to be beneficial in reducing arsenic-induced toxicity in a cell culture model (Wang et al. 2007). Stable knockdown of endogenous Nrf2 using Nrf2-shRNA rendered cells more sensitive to arsenic-induced cell death. On the other hand, pretreatment with chemicals that activate Nrf2 enhanced cell resistance to arsenic-induced cell death. The present study provides the framework of using natural compounds to activate the Nrf2-dependent protective pathway to counteract arsenic-induced damage.

In this article we report the identification of a novel class of Nrf2 activators. Oridonin, also known as rubesecensin A, is a diterpenoid purified from the Chinese medicinal herb *Rabdosia rubescens.* As one of the important traditional Chinese medicines, *R. rubescens* has been used by Chinese doctors to treat swelling of the throat, insect bites, snake bites, inflammation of the tonsils, and cancer of the esophagus, stomach, liver, prostate, and breast (Zhou et al. 2007a). The active ingredients in *R. rubescens* are rubesecensin A (oridonin) and rubesecensin B. Currently the major research focus on oridonin is in its antiproliferation and antitumor activities. The anticancer activity of oridonin is thought to rely on its ability to inhibit cell growth, reduce angiogenesis, and enhance apoptosis (Chen et al. 2005; Ikezoe et al. 2003; Liu et al. 2004, 2006; Meade-Tollin et al. 2004; Zhang et al. 2004a). Oridonin inhibits cell growth and induces apoptotic cell death in many cancer cell lines, including leukemia (NB4, HL-60, HPB-ALL, Kasumi-1), glioblastoma (U118, U138), melanoma (A375-S2), cervical carcinoma (HeLa), ovarian carcinoma (A2780, PTX10), prostate carcinoma (LNCap, Du145, PC3), breast carcinoma (MCF-7, MDA-MB231), murine fibrosarcoma (L929), and non–small-cell lung carcinoma (NCI-H520, NCI-H460, NCI-H1299) (Chen et al. 2005; Ikezoe et al. 2003; Liu et al. 2004, 2006; Zhang et al. 2004a). The reported doses needed for growth inhibition and apoptosis vary significantly among different groups using different cell lines, ranging from 0.5 μM (0.18 μg/mL) in Kasumi-1 cells to 56 μM (20.4 μg/mL) in HPB-ALL cells (Liu et al. 2006; Zhou et al. 2007b). In addition, oridonin enhances the efficacy of the cancer drug cisplatin in mouse sarcoma cells (Gao et al. 1993).

Mechanistic studies have provided a molecular basis by which oridonin inhibits cell growth and induces apoptosis. Oridonin induced p21 expression, resulting in cell cycle arrest in LNCaP and NCI-H520 cells (Ikezoe et al. 2003). Oridonin activated the caspase 3–dependent apoptotic pathway through up-regulation of Bax and down-regulation of Bcl-2, which promotes release of cytochrome c (Chen et al. 2005; Liu et al. 2006). Inhibition of telomerase activity has been reported to be another mechanism that contributes to the anticancer function of oridonin (Liu et al. 2004). Because telomerase activity is absent in normal somatic cells but is up-regulated in cancer cells or tumor tissues, this allows oridonin to specifically target abnormal tissue. In addition, total tyrosine kinase activity was reduced in response to oridonin treatment (Li et al. 2007). In addition to cancer cell lines, the efficacy of oridonin *in vivo* has been demonstrated in a colorectal carcinoma cell HT29-inoculated mouse model (Zhu et al. 2007). More significantly, a recent study using both cell culture and mouse models demonstrated that oridonin displayed a great antitumor activity specifically in acute myeloid leukemia with the t(8;21) translocation between *AML1* and *ETO* genes. Mechanistically, oridonin induced the caspase 3–dependent cleavage of the AML1–ETO fusion protein, leading to an accelerated apoptotic response (Zhou et al. 2007b).

Here, we report that oridonin belongs to a novel class of Nrf2 activators. Similar to *tert*-butylhydroquinone (tBHQ), it inhibits ubiquitination and degradation of Nrf2, resulting in stabilization of Nrf2 and activation of the Nrf2 signaling pathway. Furthermore, the chemopreventive activity of oridonin was demonstrated using a previously established arsenic-UROtsa cell model. Pretreatment of UROtsa cells with 1.4 μM oridonin significantly enhanced the cellular redox capacity, reduced formation of reactive oxygen species (ROS), and improved survival of UROtsa cells after arsenic exposure.

## Materials and Methods

### Chemicals

Most chemicals, including sodium arsenite, tBHQ, and Hoechst 33258, were from Sigma Chemical Co. (St. Louis, MO, USA). Rubescensin A (oridonin) was purchased from LKT Laboratories Inc. (St. Paul, MN, USA).

### Cell cultures

We obtained human MDA-MB-231 breast carcinoma cells from American Type Culture Collection (Manassas, VA, USA). Cells were cultured in Eagle’s minimal essential medium (MEM) supplemented with 10% fetal bovine serum (FBS), 2 mM HEPES, and 6 ng/mL bovine insulin from Sigma Chemical Co. UROtsa cells were generously provided by M.A. Sens and D. Sens (University of North Dakota). UROtsa cells were grown in Dulbecco’s modified Eagle’s medium (DMEM) enriched with 5% FBS. All mammalian cells were incubated at 37°C in a humidified incubator containing 5% CO_2_.

### Establishment of a reporter cell line

We purchased the luciferase plasmid, pGL4.22[luc2CP/Puro] from Promega (Madison, WI, USA). A 39-bp antioxidant responsive element (ARE)-containing sequence from the promoter region of the human NAD(P)H quinone oxidoreductase 1 (*NQO1*) gene was inserted into the cloning site of the luciferase plasmid. The ARE-luciferase plasmid was transfected into MDA-MB-231 cells using Lipofectamine Plus from Invitrogen (Grand Island, NY, USA) according to the manufacturer’s instructions. At 48 hr posttransfection, cells were grown in medium containing 3 μg/mL puromycin for selection. Stable cell lines were considered established once all the cells in the negative control plate were killed. Stable cell lines were continuously grown in the MEM containing 3 μg/mL puromycin. For the reporter gene assay, the ARE-luciferase stable reporter cells were seeded the day before and treated with different doses of test compounds for 24 hr. Cells were lysed in extraction buffer [0.1 M potassium phosphate and 1 mM dithiothreitol (DTT)] by freezing and thawing three times, and luciferase activities were measured in an assay buffer (25 mM glycylglycine, 15 mM magnesium sulfate, 500 μM ATP, 250 μM luciferin, and 250 μM coenzyme A) using a BioTek Synergy 2 microplate reader (Winooski, VT, USA). We performed the reporter gene assay in triplicate and calculated the mean ± SD.

### Luciferase reporter gene assay

For the dual luciferase reporter gene assay, MDA-MB-231 cells were transfected with the same ARE-luciferase plasmid along with the renilla luciferase expression plasmid, pGL4.74[hRluc/TK], from Promega. At 24 hr posttransfection, the transfected cells were treated with compounds for 24 hr, and both firefly and renilla luciferase activities were measured with the dual luciferase reporter assay system from Promega. Firefly luciferase activity was normalized to renilla luciferase activity. The experiment was carried out in triplicate and expressed as the mean ± SD.

### mRNA extraction

Total mRNA was extracted from cells using TRIZOL reagent (Invitrogen), and equal amounts of RNA were reverse-transcripted to cDNA using the Transcriptor First Strand cDNA synthesis Kit (Roche, Indianapolis, IN, USA). The PCR condition, as well as Taqman probes and primers for *Nrf2, NQO1*, heme oxygenase-1 (*HO-1*), and *GAPDH* were reported previously (Wang et al. 2007). Briefly, we obtained the following Taqman probes from the universal probe library (Roche): *hNrf2* (#70), *hNQO1* (#87), *hHO-1* (#25), and *hGAPDH* (#25). The following primers were synthesized by Integrated DNA Technologies (Coralville, IA, USA): *hNrf2*: forward (acacggtccacagctcatc) and reverse (tgtcaatcaaatccatgtcctg); *hNQO1*: forward (atgtatgacaaaggacccttcc) and reverse (tcccttgcagagagtacatgg); *hHO-1*: forward (aactttcagaagggccaggt) and reverse (ctgggctctccttgttgc); and *hGAPDH*: forward (ctgacttcaacagcgacacc) and reverse (tgctgtagccaaattcgttgt).

### Real-time reverse transcriptase-polymerase chain reaction (RT-PCR)

The real-time PCR condition was as follows: one cycle of initial denaturation (95°C for 10 min), 40 cycles of amplification (95°C for 10 sec and 60°C for 20 sec), and a cooling period (50°C for 5 sec). The data presented are relative mRNA levels normalized to *GAPDH*, and the value from the untreated cells was set as 1. We used triplicate samples to determine the mean ± SD.

### Antibodies and immunoblot analysis

We purchased the antibodies for Nrf2, Keap1, and β-actin from Santa Cruz Biotechnology (Santa Cruz, CA, USA). Cells were lysed in a sample buffer [50 mM Tris-HCl (pH 6.8), 2% SDS, 10% glycerol, 100 mM DTT, 0.1% bromophenol blue]. After sonication, cell lysates were electrophoresed through an SDS-polyacrylamide gel and subjected to immunoblot analysis. For detection of the ubiquitinated Nrf2 *in vivo*, cells were transfected with expression vectors for hemagglutinin (HA)-ubiquitin, Keap1, and Gal4-Neh2 (the N-terminal domain of Nrf2 containing the ubiquitin conjugating sites). The transfected cells were either left untreated or treated with chemicals along with 10 μM MG132 (Sigma Chemical Co.) for 4 hr. Cells were lysed by boiling in a buffer containing 2% SDS, 150 mM NaCl, 10 mM Tris-HCl, and 1 mM DTT. These lysates were then diluted 5-fold in buffer lacking SDS and incubated with anti-Nrf2 or anti-Keap1 antibodies. Immunoprecipitated proteins were analyzed by immunoblot with antibodies directed against the HA epitope (Zhang and Hannink 2003).

### Ubiquitination assay

To detect endogenous Nrf2 ubiquitination, the UROtsa cells were treated with 10 μM MG132 and lysed and diluted in the same way. Nrf2 was immunoprecipitated with an anti-Nrf2 antibody and subjected to immunoblot analysis with an antiubiquitin antibody (Sigma Chemical Co.).

### Protein half-life measurement

To measure the half-life of Nrf2, cells were either left untreated or treated with oridonin for 4 hr. To block protein synthesis, we added 50 μM cycloheximide. Total cell lysates were collected at different time points and subjected to immunoblot analysis with an anti-Nrf2 antibody. The relative intensity of bands was quantified by the ChemiDoc CRS gel documentation system and Quantity One software from BioRad (Hercules, CA, USA).

### Transient transfection of siRNA and measurement of glutathione concentration

We purchased Nrf2-siRNA and the control siRNA from Qiagen (Valencia, CA, USA). Transient transfection of siRNA was performed using HiPerFect Transfection Reagent according to the manufacturer’s protocol (Qiagen). Intracellular glutathione concentration was measured using the QuantiChrom glutathione assay kit from BioAssay Systems (Hayward, CA, USA). All the procedures were carried out according to the manufacturer’s instructions.

### ROS detection

For detection of ROS, we pretreated cells with 1.4 μM oridonin for 24 hr, followed by As(III) treatment or As(III) plus oridonin cotreatment for another 24 hr. ROS levels were measured using dichlorofluorescein (Sigma Chemical Co., 10 μg/mL final concentration) and flow cytometry.

### Detection of cell viability

Cell viability was measured by 3-(4, 5-dimethylthiazol-2-yl)-2,5-diphenyl-2H-tetrazolium bromide (MTT) (Wang et al. 2007) and by colony formation assays. We performed colony formation assays in 35-mm plates with 200 UROtsa cells. Attached cells were left untreated or were treated with oridonin for 24 hr, followed by treatment with different doses of As(III) for another 48 hr. After exposure, the medium was replaced with fresh medium, and cells were incubated for 12–14 days. The cells were then fixed and stained with crystal violet (0.5% in 95% ethanol), and the colonies in each plate were counted.

### Detection of cell death

For detection of apoptotic cell death, we used two different methods: *a*) Annexin V-FITC apoptosis detection (Sigma Chemical Co.), and *b*) Hoechst staining (1 μg/mL) for detection of the condensed nuclei. All experiments were conducted in triplicate and expressed as mean ± SD. The statistical significance was determined by the Student’s *t*-test.

## Results

### Identification of oridonin as an Nrf2 activator

**Figure 1:**
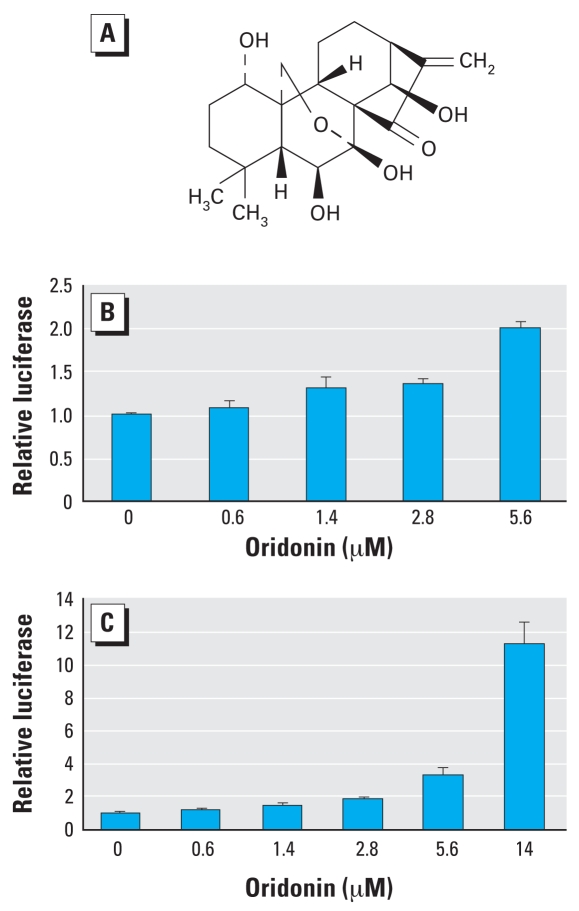
(*A*) Structure of the diterpenoid oridonin. (*B, C*) Luciferase reporter gene assays in MDA-MB-231 cells expressing ARE-luciferase. (*B*) Luciferase activity showing oridonin as an Nrf2 activator using a high-throughput screening system. The stable MDA-MB-231 cells expressing ARE-luciferase were seeded in 96-well plates; cells were grown to 90% confluence and treated with oridonin for 24 hr before analysis of luciferase activity. (*C*) Luciferase activity in MDA-MB-231 cells cotransfected with a plasmid containing an ARE-luciferase reporter gene and a plasmid encoding renilla luciferase driven by the herpes simplex virus thymidine kinase promoter. The transfected cells were treated with oridonin for 24 hr prior to measurement of firefly and renilla luciferase activities in cell lysates. All luciferase reporter gene assays were run in triplicate and expressed as mean ± SD.

Using the stable ARE luciferase reporter cell line derived from MDA-MB-231 cells combined with a 96-well high-throughput screening system established in our laboratory, we identified a novel Nrf2 activator that belongs to the class of diterpenoids (Figure 1A). The MDA-MB-231 cell line was used to show Nrf2 activation for two reasons: first, MDA-MB-231 cells can be easily transfected; and second, the Nrf2 pathway is most sensitive in this cell line in response to Nrf2-inducers. Oridonin induced transcription of the ARE-dependent luciferase gene in a dose-dependent manner in this stable cell line (Figure 1B). To confirm oridonin activation of Nrf2 using the high-throughput screening method, we also performed a dual luciferase reporter gene assay in which we included a renilla luciferase gene as an internal control for transfection efficiency and for toxicity induced during oridonin exposure. Consistent with the data obtained from the high-throughput screening, oridonin induced the ARE-dependent luciferase activity in a dose-dependent manner (Figure 1C). Slight induction (1.5-fold) was observed at as low as 1.4 μM and reached maximum induction (11.3-fold) at 14 μM. There was no obvious toxicity at 14 μM, as judged by cell morphology and renilla luciferase activity.

### Oridonin activated the ARE-dependent response primarily through up-regulation of the Nrf2 protein level

**Figure 2:**
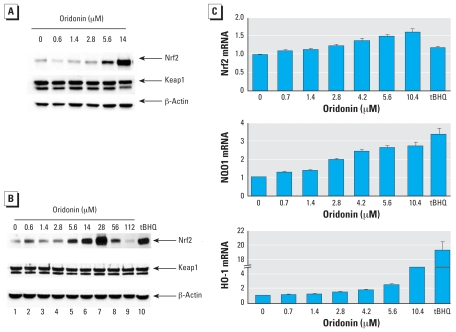
Effects of oridonin on MDA-MB-231 cells. (*A*) An aliquot of cell lysates from the dual luciferase reporter gene assay was subjected to immunoblot analysis with anti-Nrf2, anti-Keap1, and anti-β-actin. (*B*) Total cell lysates from MDA-MB-231 cells treated with oridonin for 24 hr were subjected to immunoblot analysis with anti-Nrf2, anti-Keap1, and anti-β-actin antibodies. (*C*) mRNA from similarly treated cells was extracted and reverse transcribed into cDNA prior to real-time PCR analysis for detection of mRNA for *Nrf2* (top), *NQO1* (center), and *HO-1* (bottom).

Previous studies have demonstrated that ARE-dependent reporter gene activity correlated very well with the protein level of Nrf2. Therefore, we used the same cell lysates from the dual luciferase reporter gene assay for immunoblot analysis for detection of Nrf2, Keap1, and β-actin. Although the Keap1 levels remained constant, oridonin enhanced the levels of Nrf2 protein in a dose-dependent manner, with the highest induction at 14 μM (Figure 2A). During the reporter gene assay, any doses > 14 μM caused marked toxicity, as indicated by an increased number of rounded and floating cells. A large body of literature indicates that the antitumor activity of oridonin relies on its ability to inhibit cell growth and to induce cell death (Zhou et al. 2007a). Because Nrf2 regulates a cellular survival response, we envisioned that treatment with high doses of oridonin could inhibit Nrf2, allowing cells to undergo cell death. Therefore, we tested Nrf2 protein levels in response to high doses of oridonin. After treatment of MDA-MB-231 cells with different doses of oridonin for 24 hr, we collected all cells, including floating cells. Equal amounts of proteins were subjected to immunoblot analysis with Nrf2, Keap1, and β-actin antibodies. Interestingly, at doses > 28 μM, Nrf2 protein levels decreased in a dose-dependent manner, whereas the expression of Keap1 and β-actin had no significant change (Figure 2B, lanes 7–9). Previously, it has been demonstrated that Nrf2 activators, including tBHQ, induce the Nrf2 signaling pathway primarily through stabilization of the Nrf2 protein, rather than up-regulation of its mRNA (Nguyen et al. 2003).

Next, we measured mRNA expression of *Nrf2* and its target genes, *NQO1* and *HO-1*, in response to oridonin using real-time RT-PCR. Nrf2 mRNA increased slightly in a dose-dependent manner in response to oridonin, whereas tBHQ had no effect (Figure 2C, top panel). As expected, mRNA of *NQO1* and *HO-1* were induced significantly by oridonin in a dose-dependent manner (Figure 2C, center and bottom panels). These results demonstrate that oridonin is able to induce the Nrf2 signaling pathway mainly through up-regulation of Nrf2 at the protein level.

### Oridonin blocked Nrf2 ubiquitination and enhanced Keap1 ubiquitination

**Figure 3:**
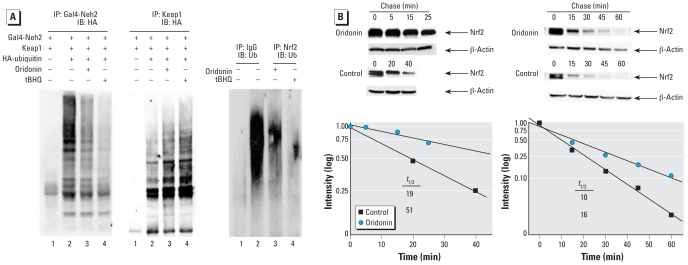
Effects of oridonin on Nrf2 and Keap1 ubiquitination and protein half-life in MDA-MB-231 cells or UROtsa cells. Abbreviations: IB, immunoblot; IP, immunoprecipitation; *t*_1/2_, half-life; Ub, ubiquitin. (*A*) MDA-MB-231 cells were cotransfected with expression vectors for HA-ubiquitin, a Gal4–Neh2 fusion protein, and Keap1; the transfected cells were left untreated or treated with 8.4 μM oridonin or 100 μM tBHQ for 4 hr, along with 10 μM MG132. Cells were lysed in 2% SDS and immediately heated. Anti-Gal4 (left) and anti-Keap1 (center) immunoprecipitates analyzed by immunoblot with anti-HA antibodies for detection of the ubiquitin-conjugated Neh2 or Keap1. (Right) Ubiquitination of endogenous Nrf2 assessed in UROtsa cells treated with DMSO, 8.4 μM oridonin, or 100 μM tBHQ for 4 hr, along with 10 μM MG132; Nrf2 was immunoprecipitated with an anti-Nrf2 antibody, and ubiquitinated Nrf2 was detected with an anti-ubiquitin antibody. (*B*) Protein half-life in MDA-MB-231 cells (left) and UROtsa cells (right) left untreated or treated with 8.4 μM oridonin for 4 hr. Cycloheximide (50 μM) was added to block protein synthesis. Cells were lysed at the indicated time points, and lysates were subjected for immunoblot analysis with anti-Nrf2 and anti-β-actin antibodies (top). Intensity of the bands was quantified using Quantity One software (bottom). In *B*, the bottom left and right graphs represent the bands in the top left and right, respectively.

tBHQ enhances the Nrf2 protein level by interfering with the Keap1-dependent ubiquitin conjugation process. Therefore, we tested the ability of oridonin in modulating Nrf2 ubiquitination. For this assay, we used Gal4-Neh2, a model fusion protein previously used for the Nrf2 ubiquitination test (Zhang and Hannink 2003). In a manner similar to tBHQ, oridonin suppressed Nrf2 ubiquitination (Figure 3A, left panel).

Furthermore, Zhang et al. (2005) showed that tBHQ caused a shift of ubiquitination from the substrate Nrf2 to the substrate adaptor Keap1. As with tBHQ, oridonin treatment was also effective in enhancing ubiquitination of Keap1 (Figure 3A, center panel). These results demonstrate that oridonin can induce a shift of ubiquitination from Nrf2 to Keap1. One of the major roles for ubiquitin conjugation onto a protein is to target the protein for 26S proteasome-mediated degradation. Next, we measured the half-life of Nrf2 in the absence or presence of oridonin. Half-life of the endogenous Nrf2 protein in MDA-MB-231 cells was 19 min, whereas treatment with oridonin increased the half-life to 51 min (Figure 3B, left panel). Taken together, these results indicate that oridonin activates the Nrf2 pathway by inhibiting ubiquitination and degradation of Nrf2, leading to an increase in Nrf2 protein level and activation of the Nrf2-dependent response.

### Efficacy of oridonin in protecting against As(III)-induced toxicity

To test the feasibility of using oridonin as a chemopreventive compound to elicit the Nrf2-mediated protective response to defend against environmental insults, we used the UROtsa cell line, an established model system for arsenic toxicity. First, we determined activation of the Nrf2 pathway by oridonin in this cell line. Ubiquitination of endogenous Nrf2 in UROtsa cells was blocked by oridonin or tBHQ treatment (Figure 3A, right panel). Consistent with a decrease in ubiquitination of Nrf2 in response to oridonin, the half life of Nrf2 was increased from 10 min in the untreated condition to 16 min in response to oridonin treatment (Figure 3B, right panel).

**Figure 4:**
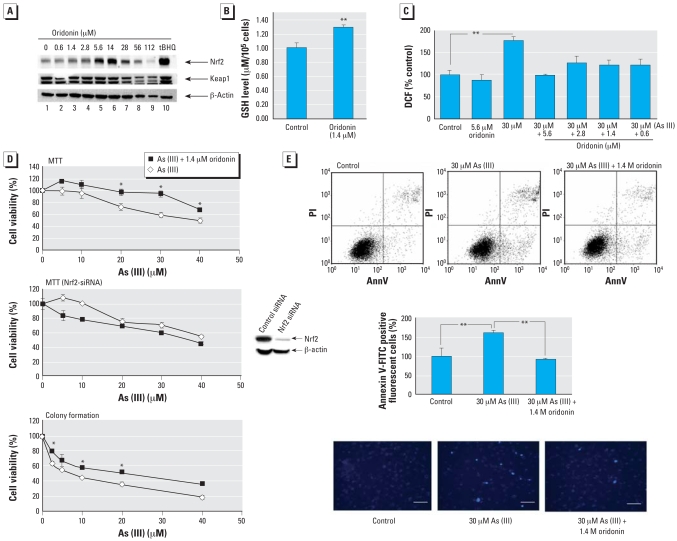
Effect of oridonin on UROtsa cells. Abbreviations: AnnV, Annexin V; DCF, 2′,7′-dichlorodihydrofluorescein; PI, propidium iodide. (*A*) Immunoblot showing Nrf2, Keap1, and β-actin in UROtsa cells treated with oridonin for 24 hr; cell lysates were collected and subjected to immunoblot analysis with anti-Nrf2, anti-Keap1, and anti-β-actin antibodies. (*B*) Intracellular glutathione concentrations in UROtsa cells either untreated (control) or treated with 1.4 μM oridonin. Concentrations were measured using the QuantiChrom glutathione assay kit; values shown are the mean ± SD of experiments run in triplicate. (*C*) ROS analysis in UROtsa cells untreated or pretreated with oridonin for 24 hr and then further treated with As(III) or As(III) plus oridonin, respectively, for another 24 hr. ROS were measured by dichlorofluorescein/flow cytometry; values shown are the mean ± SD of experiments run in triplicate. (*D*) Cell survival in UROtsa cells untreated or pretreated with 1.4 μM oridonin for 24 hr and then treated with As(III) in the absence or presence of 1.4 μM oridonin for another 48 hr; values shown are the mean ± SD of experiments run in triplicate. (*D,* top) Cell survival measured by the MTT assay. (*D,* center, at right) Immunoblot analysis showing Nrf2 protein levels in UROtsa cells transfected with control siRNA or Nrf2-siRNA for 48 hr; Nrf2 protein levels were assessed by immunoblot analysis with an anti-Nrf2 antibody to con-firm knockdown of Nrf2 expression. (*D,* center) Cell survival in Nrf2-siRNA transfected cells at 48 hr posttransfection measured by the MTT assay; 200 cells in 35-mm plates were pretreated and cotreated in the same manner as in the MTT assay. (*D*, bottom) Cell survival measured by the colony formation assay. Values shown are the mean ± SD of experiments run in triplicate. (*E*) Apoptotic cell death in UROtsa cells untreated or pretreated with 1.4 μM oridonin for 24 hr and then treated with As(III) in the absence or presence of 1.4 μM oridonin for another 48 hr. Apoptotic cell death was detected using Annexin V-FITC and flow cytometry; the mean ± SD was calculated from experiments run in triplicate (center). (*E*, bottom) Apoptois in UROtsa cells grown on cover slides were pretreated and cotreated in the same way. Apoptotic cells were visualized by condensed nuclei using Hoechst staining; bars = 25 μm. The experiment was repeated, and similar results were obtained. *p < 0.05. **p < 0.01.

Next, we determined the oridonin dose range that induces Nrf2 protein in UROtsa cells. Compared with MDA-MB-231 cells (Figure 2B, lanes 2–7), UROtsa cells had a narrow range of Nrf2 induction, from 1.4 to 14μM (Figure 4A, lanes 3–6). Doses > 14 μM appeared to be toxic, and induction of Nrf2 was decreased (Figure 4A, lanes 7–9). At 56 or 112 μg/mL, there was a decrease even in the level of β-actin due to cytotoxicity (Figure 4A, lanes 8 and 9). Nevertheless, reduction of the Nrf2 protein was significantly more substantial, indicating that reduction of Nrf2 at high doses may not be due solely to reduced cell number. Based on this result, we chose a low dose of oridonin (0.5 μg/mL) for the protection assays.

One of the major functions of Nrf2 is to regulate an antioxidant response by up-regulating intracellular antioxidants and genes such as *GCS* and the *xCT* cysteine antiporter that encode key enzymes in the synthesis of glutathione. To confirm activation of the Nrf2-dependent response by 1.4 μM oridonin, we compared the intracellular glutathione level in the oridonin-treated cells with that in untreated cells. Oridonin treatment resulted in a significant increase in the glutathione level (Figure 4B). Thus, oridonin is able to augment the cellular redox capacity, which is the key mechanism in suppressing oxidative stress–induced damage by environmental insults. In the protection assays, we used sodium arsenite [As(III)] to treat UROtsa cells. We measured the ability of oridonin to alleviate As(III)-induced ROS (Figure 4C). Treatment with 30 μM As(III) for 24 hr increased the level of ROS significantly, whereas 5.6 μM oridonin itself had no effect. Pretreatment of cells with several doses of oridonin for 24 hr and further cotreatment with As(III) for an additional 24 hr resulted in a significant reduction of ROS levels, especially with 5.6 μM oridonin. These data clearly demonstrate the efficacy of oridonin in suppressing oxidative stress imposed by As(III) exposure.

Finally, we assessed the effectiveness of oridonin in protecting cells from acute cell death in response to As(III). UROtsa cells were left untreated or were pretreated with 1.4 μM oridonin. After a 24-hr pretreatment period, several doses of As(III) were added to both groups and incubated for an additional 48 hr before measuring total cell death using both MTT and colony formation assays. Pretreatment followed by cotreatment with oridonin significantly improved cell survival as judged by the MTT assay (Figure 4D, top panel) and the colony formation assay (Figure 4D, bottom panel). To confirm that protection against As(III)-induced cell death was attributed to the activation of Nrf2 by oridonin, the MTT assay was performed in UROtsa cells that were treated with Nrf2-siRNA for 48 hr. Immunoblot analysis confirmed the effectiveness of Nrf2-siRNA in reducing Nrf2 expression (Figure 4D, center panel). Inhibition of Nrf2 expression in UROtsa cells reverted the MTT curve [i.e., oridonin lost its protection against As(III) toxicity] and aggravated As(III)-induced cell death (Figure 4D, center panel). This result demonstrates that oridonin-mediated protection requires activation of the Nrf2 pathway.

Apoptotic cell death was quantified using Annexin V-FITC/flow cytometry. Treatment with 30 μM As(III) for 48 hr increased the percentage of apoptotic cells, whereas pre-treatment followed by cotreatment with 1.4 μM oridonin reduced apoptotic cell death to a level comparable to the untreated cells (Figure 4E, top and center panels). Apoptotic cell death was not increased by 1.4 μM oridonin alone (data not shown). We used Hoechst staining to detect condensed chromosomes in the apoptotic cells. The number of positive-stained cells increased after treatment with 30 μM As(III), whereas pre-treatment followed by cotreatment with oridonin markedly reduced the number of apoptotic cells (Figure 4E, bottom panel). Together, these results demonstrate that a low dose of oridonin is able to protect cells from As(III)-induced damage, as illustrated by reduced ROS and increased survival in response to As(III).

## Discussion

The pivotal role of Nrf2 in chemoprevention has clearly been shown in Nrf2 null mice. These mice express lower basal levels of the Nrf2 target genes such as *NQO1*, *GST*, *GCS*, UDP-glucuronosyltransferase, glutathione peroxidase-2, and *HO-1* (Chan and Kwong 2000; Cho et al. 2002; Hayes et al. 2000; Kwak et al. 2001; McMahon et al. 2001). As a consequence, these mice are more susceptible to toxic and carcinogenic challenges such as butylated hydroxytoluene, benzo[*a*]pyrene, diesel exhaust, aflatoxin B_1_, *N*-nitrosobutyl (4-hydroxybutyl) amine, pentachlorophenol, acetaminophen, ovalbumin, cigarette smoke, and 4-vinyl cyclohexene diepoxide (Aoki et al. 2001; Chan et al. 2001; Chan and Kan 1999; Enomoto et al. 2001; Hu et al. 2006; Iida et al. 2004; Iizuka et al. 2005; Ramos-Gomez et al. 2001; Rangasamy et al. 2005; Umemura et al. 2006). These results provide the basis for chemopreventive intervention targeting the Nrf2 signaling pathway. Many previously indentified, naturally occurring compounds, including sulforaphane, epigallocatechin-3-gallate, caffeic acid phenethyl ester, and curcumin, have proved to be Nrf2 activators, which further implies the importance of Nrf2 in chemoprevention (Jeong et al. 2006; Nishinaka et al. 2007; Zhang 2006). Identification, validation, and optimization of new Nrf2 activators are essential for the development of effective dietary supplements or therapeutic drugs that can be used to boost the Nrf2-dependent adaptive system to protect humans from various environmental insults.

Oridonin represents a novel class of Nrf2 activators that has not been demonstrated previously. Mechanistic studies presented here indicate that oridonin induced the Nrf2-dependent response primarily by enhancing the Nrf2 protein level. The increase in the Nrf2 protein level in response to oridonin is attributed mainly to the stabilization of Nrf2, with minor contribution from increased mRNA. Similar to tBHQ, oridonin is able to block ubiquitination and degradation of Nrf2, resulting in the prolonged half-life of Nrf2. Furthermore, we demonstrated the effectiveness of a low dose of oridonin (1.4 μM) in eliciting the Nrf2-dependent cytoprotective response in an As(III)-toxicity model. Low doses of oridonin are able to enhance the cellular reducing capacity by significantly elevating the reduced glutathione level, thus inhibiting the formation of ROS, resulting in increased survival in response to As(III) exposure. Furthermore, glutathione is able to conjugate arsenic to facilitate arsenic excretion, thereby reducing As(III) toxicity (Shinkai et al. 2006). In addition to *GCS*, which modulates intracellular glutathione levels, other Nrf2 downstream genes, including *GST*, UDP-glucuronosyl transferase, and multidrug resistance proteins, also contribute to the Nrf2-mediated protection against arsenic toxicity (Hayashi et al. 2003; Kobayashi and Yamamoto 2006; Maher et al. 2005; Vernhet et al. 2001; Xu et al. 2005; Zhang 2006). Although the present study shows only the protection of oridonin against acute As(III) toxicity, oridonin certainly can be applied to other toxic and carcinogenic chemicals, because oridonin induces the well-characterized Nrf2-dependent defensive response.

This cell-based study provides evidence that low-dose oridonin can be used as a chemopreventive compound that specifically targets Nrf2. Further studies on the chemo-preventive activity of oridonin in animal models are needed. If oridonin is shown to have great chemopreventive potential, then it has a great economic advantage because it can easily be extracted from *Rabdosia rubescens* “the Chinese grass.” In addition, identification of diterpenoids as a new class of Nrf2 activators will broaden the choice for new chemopreventive compounds. Moreover, the diterpenoid structure can serve as a scaffold for the development of chemopreventive drugs. Identification of naturally occurring diterpenoids or synthetic optimization of the diterpenoid oridonin, which potently and specifically induce the Nrf2 signaling pathway, will greatly improve the efficacy of chemopreventive drugs and decrease side effects, which will have a profound impact on human health.

High doses of oridonin promote anti-cancer activity by causing cell cycle arrest, inhibiting proliferation, and inducing apoptotic cell death. The dose range needed for oridonin to exhibit anticancer activities in these studies, conducted by different laboratories with a variety of cancer cell lines, is very broad, with 100-fold differences. Although this may be due partially to the purity of oridonin used among groups, it largely indicates a difference in sensitivity of cancer cells to the oridonin-induced apoptotic response. In the present study, the effect of oridonin in inducing the Nrf2 protein level was assessed in two different cell lines, breast carcinoma MDA-MB-231 cells and immortalized but non-transformed bladder urothelium UROtsa cells. UROtsa cells showed a narrower window of Nrf2 induction in response to different doses of oridonin. It is interesting to note that oridonin induced Nrf2 protein and reporter gene activity in a dose-dependent manner to a certain point at which the Nrf2 protein level and the reporter gene activity dropped suddenly. We observed an initial decrease in Nrf2 protein in MDA-MB-231 cells and UROtsa cells at 56 μM and 28 μM oridonin, respectively. This decrease in Nrf2 protein level in response to high doses of oridonin is not due solely to cell toxicity because Keap1 or β-actin levels decreased only slightly. Based on the important role of Nrf2 in cell survival, it is conceivable that Nrf2 has to be repressed before the execution of cell death. In support of this notion, Nrf2 has been reported as a substrate of caspase 3 (Ohtsubo et al. 1999). The cleavage sites in Nrf2 for caspase 3 have been identified. Based on the Nrf2 induction profile, it is remarkable that oridonin functions as a chemopreventive compound at low doses by activating the Nrf2 cytoprotective pathway, whereas at high doses, it activates apoptotic cell death and concomitantly inhibits the Nrf2-dependent survival pathway. Further studies are needed to understand the molecular events that cause the switch between life and death.
